# NAD^+^ attenuates experimental autoimmune encephalomyelitis through induction of CD11b^+^ gr-1^+^ myeloid-derived suppressor cells

**DOI:** 10.1042/BSR20200353

**Published:** 2020-04-24

**Authors:** Jin-Li Wang, Bin Li, Guo-Jun Tan, Xiao-Li Gai, Jun-Na Xing, Jue-Qiong Wang, Mo-Yuan Quan, Ning Zhang, Li Guo

**Affiliations:** Department of Neurology, The Second Hospital of Hebei Medical University, Key Laboratory of Hebei Neurology, No. 215 Heping Road, Shijiazhuang 050000, Hebei, China

**Keywords:** experimental autoimmune encephalomyelitis, inflammatory cells, multiple sclerosis, myeloid-derived suppressor cells, NAD+

## Abstract

Objective: To investigate the effects of nicotinamide adenine dinucleotide (NAD^+^) on the pathogenesis of the animal model for multiple sclerosis (MS)-experimental autoimmune encephalomyelitis (EAE).

Methods: EAE model was induced by myelin oligodendrocyte protein (MOG 35-55). Clinical scores of EAE were measured in mice with or without NAD^+^ treatment. Hematoxylin and Eosin (HE) and Luxol Fast Blue (LFB) staining were performed to assess inflammation and demyelination, respectively. Expressions of target proteins were measured by Western blot. The numbers of myeloid-derived suppressor cells (MDSCs) were measured by immunofluorescent staining and flow cytometry. Enzyme-linked immunosorbent assay (ELISA) was used to measure the expressions of inflammatory cytokine in serum.

Results: NAD^+^ treatment could decrease inflammatory cells and demyelination foci, attenuate the clinical scores of EAE and slightly delay disease onset. Western blot showed that NAD^+^ treatment up-regulated the expression of phosphorylated-STAT6 (p-STAT6) and SIRT1. Besides, NAD^+^ treatment up-regulated the expression of p-IκB and down-regulated the expression of p-NF-κB. In addition, NAD^+^ treatment could increase the numbers of CD11b^+^ gr-1^+^ MDSCs and the expression of Arginase-1. Moreover, NAD^+^ treatment up-regulated the expressions of IL-13 and down-regulated the expression of IFN-γ and IL-17.

Conclusions: The present study demonstrated that NAD^+^ treatment may induce the CD11b^+^ gr-1^+^ MDSCs to attenuate EAE via activating the phosphorylation of STAT6 expression. Therefore, NAD^+^ should be considered as a potential novel therapeutic strategy for MS.

## Introduction

Multiple sclerosis (MS) is a complex chronic autoimmune disease, involving central nervous system (CNS) demyelination and inflammation [1]. There are approximately 2.5 million people suffering from MS all over the world, while in Western countries, 1 in 1000 people suffer from MS [2,3]. At present, the treatment of MS is mainly anti-inflammatory, but it is only effective in relapse and remission period [4]. In addition, some of these agents (such as Tysabri) may have severe immunosuppressive side effects [5]. Thus, developing novel therapeutic strategies has been an urgent problem for patients with MS.

Experimental autoimmune encephalomyelitis (EAE) is the most widely used mouse model of MS, which is mediated by cells of the innate immune system and autoimmune CD4^+^ T cells [6]. The histopathological and clinical similarities between EAE and MS enable the findings obtained from EAE model to be extrapolated to MS patients [7]. Recent studies have shown that myeloid-derived suppressor cells (MDSCs)—a heterogeneous population of myeloid cells plays important role in shaping T-cell responses [1,8]. MDSCs are usually identified in mice by the co-expression of the CD11b and Gr-1 surface markers. MDSCs are reported to attenuate the clinical course of EAE [9,10]. In addition, study has found that STAT6 could promoted the expansion of MDSCs in the lamina propria and spleen of ApcMin/^+^ mice [11]. However, the regulatory effect of STAT6 on MDSCs in EAE remains unclear.

SIRT1 is a nicotinamide adenine dinucleotide (NAD^+^)-dependent protein deacetylase that catalyzes the removal of acetyl groups from a variety of protein substrates [12]. Previous study showed that SIRT1 induction can attenuate the course of EAE [13]. However, the underlying mechanism has not been well elucidated. Interestingly, Liu et al. [14] found that SIRT1 exerting an anti-acute gouty arthritis effect by activating the PI3K/Akt/STAT6 pathway. Combining these published evidences, we speculated that SIRT1 induction may induce the CD11b^+^ gr-1^+^ MDSCs to attenuate EAE by STAT6.

Thus, the aim of the present study was to investigate the effects of NAD^+^ on the pathogenesis of EAE. We established an EAE model and investigate the possible mechanisms of NAD^+^ in attenuating the course of EAE from the perspective of MDSCs for the first time. We proposed a novel molecular mechanism of the NAD^+^ in the improvement of EAE, in which NAD^+^ treatment may induce MDSCs to attenuate EAE via activating the phosphorylation of STAT6 expression.

## Materials and methods

### Animals

C57BL/6 female mice (6–8 weeks, weighing 20 ± 2 g) were purchased from Beijing Vital River Laboratory Animal Technology Co. Ltd (Beijing, China). They were housed in groups of four to six per cage in laminar flow hoods in a pathogen-free environment (55 ± 10% humidity, 22 ± 2°C and 12–12 h/light–dark cycle) with free access to standard laboratory water and diet. The animals were acclimatized for 1 week before the experiments. All experiments including animal experiments were carried out at the Hebei Medical University. The experiments were performed in accordance with the guidelines for animal care and the experimental protocols were approved by Ethics Review Committee of the Hebei Medical University (number 20191018). All animals were anesthetized by sodium pentobarbital injection and killed by asphyxiation in a carbon dioxide chamber.

### Induction of EAE

EAE model was induced following the procedure as described previously [15,16]. The mice received a subcutaneous injection at four sites on the flanks with 200 µg of mouse myelin oligodendrocyte protein (MOG 35-55) (XIAN LINTAI Bioscience and Technology Co., Xi’an, China) in 0.1 ml phosphate buffer saline (PBS), emulsified in 0.1 ml complete Freund’s adjuvant (CFA) (Difco, St. Louis, MO) containing 400 µg of *Mycobacterium tuberculosis H37Ra*. Furthermore, an intraperitoneal injection of 0.5 ml pertussis toxin (PT) (List Biological Labs, Inc, San Josa, CA) was given at the beginning of the induction and 48 h later.

We scored the behavioral deficits daily for all of the mice in a double-blind manner in a weaver method with 15-point behavioral scoring scale as described previously [17,18]. The 15-point scale was the sum of the disease state for the tail (scored 0–2) and all the four limbs (scored 0–3). For the tail, a score of 0 represented no signs, 1 represented a half paralyzed tail and 2 represented a fully paralyzed tail. For the four limbs, each assessed separately, 0 signified no signs, 1 represented a weak or altered gait, 2 represented paresis, while a score of 3 denoted a fully paralyzed limb. Thus, a fully quadriplegic animal would attain a score of 14 and mortality would be given a score of 15.

The NAD was administered by referring to previous reports [13,19]. NAD^+^ (250 mg/kg, diluted in PBS) treatment (NAD^+^ group) of EAE mice was started at the day of inoculation and given everyday until end of study. EAE model group was subcutaneously administered PBS (EAE model group) daily from the day of inoculation.

### Hematoxylin and Eosin staining

Mice spinal cords or spleen were fixed, embedded in paraffin. Sections were dewaxed in xylene and rehydrated in a series of alcohol solutions. After washing in distilled water for a short time, the sections were stained in Harris Hematoxylin solution for 5 min, washed in tap water and counterstained in Eosin–Phloxine solution for 2 min.

### Luxol Fast Blue staining

Luxol Fast Blue (LFB) staining was performed as previously reported [20]. Briefly, tissue slides were rehydrated and incubated in 0.1% LFB solution (60°C, 2 h). Sections were processed using 95 and 70% ethanol to remove excess stain and observe myelin staining. To increase the color contrast of the myelin, gray matter was distinguished from white matter by 0.05% lithium carbonate. Sections were then washed with distilled water, dehydrated quickly with 100% ethanol and cleared in dimethyl benzene, covered and observed under a microscope (Olympus, Japan). Scores between 0 and 3 were used to evaluate demyelination. A score of 3 was defined as totally normal myelin sheath, whereas 0 was defined as complete demyelination. Scores of 1 or 2 were defined as one-third or two-thirds myelination of the corpus callosum, respectively.

### Immunofluorescent staining

Tissue sections were dewaxed by xylene and dehydrated by graded alcohol. After washing with distilled water, tissue sections were heated by high pressure in Ethylene Diamine Tetraacetic Acid (EDTA) antigen repair buffer (pH 8.0) for antigen retrieval. After the slices were cooled at room temperature, sections were incubated with primary antibodies (1:500, 4°C, overnight) and secondary antibodies (37°C, 50 min), respectively. Then paraffin sections were incubated with 4′,6-Diamidino-2-phenylindole (DAPI, Beyotime Biotechnology, China) for 10 min in the dark. Finally, paraffin sections were sealed in anti-fluorescence. Images were acquired on a laser scanning confocal microscope FV12-IXCOV (OLYMPUS, Japan). Green fluorescence represented CD11b and red fluorescence represented Gr-1. The mean optical density was calculated by Image-J to analyze the positive expression.

### Antibodies and flow cytometry

The numbers of CD11b^+^ gr-1^+^ MDSCs were measured by flow cytometry using anti-CD11b and anti-Gr-1 antibodies. Anti-CD11b and anti-Gr-1 were purchased from eBioscience. Samples were acquired using a BD LSR II Fortessa instrument and analyzed with FlowJo software (TreeStar). All samples are analyzed in single. In each experiment, at least three to four samples were analyzed for each group.

### Enzyme-linked immunosorbent assay

The antibodies against IFN-γ, IL-13 and arginase-1 in blood serum samples were investigated by a commercial Enzyme-linked immunosorbent assay (ELISA) test kit (Biochek, Holland). The test procedure was performed according to the manufacturer’s protocol. The absorbance at 450 nm was read by an ELISA reader spectrophotometer (ELX800, Bio-Tek Inst Inc, U.S.A.). The presence of antibody in blood serum samples was determined by calculating sample to positive control ratio. The result was accepted as positive when samples had S/P ratio of greater than 0.50. If the S/P ratio of samples were less than 0.50, it was considered as negative.

### Western blot

Total proteins were extracted from the mouse spinal cords, and BCA method was used for protein quantification. Equal amounts of proteins (20 µg) of each sample were separated by sodium dodecyl sulfate/polyacrylamide gel electrophoresis and then transferred on to the polyvinylidene fluoride (PVDF) membranes (Millipore, U.S.A.). PVDF membrane was blocked in 5% skim milk at 37°C for 2 h, and subsequently incubated at 4°C overnight with antibodies against phosphorylated-STAT6 (p-STAT6), t-STAT6, arginase-1, IL-17, IFN-γ or β-ACTIN/GAPDH (all in 1:1000 dilutions, Cell Signaling Technology, Danvers, Massachusetts). Next, PVDF membrane was incubated with corresponding horseradish peroxidase–conjugated (HRP)-linked secondary IgG antibodies (1:10000 dilutions, Santa Cruz, U.S.A.) for 2 h at room temperature. The signal was detected with a Super Signal Protein Detection kit (Pierce Biotechnology, U.S.A.).

### Statistical analysis

All statistical analyses were performed by using SPSS version 22.0 (SPSS Institute, IL, U.S.A.). Results from each experiment were expressed as the mean ± standard deviation of three separate experiments. Student’s *t* test was used for comparison between the two groups. Statistical significance was set at *P*<0.05.

## Results

### NAD^+^ treatment attenuated EAE

**Figure 1:**
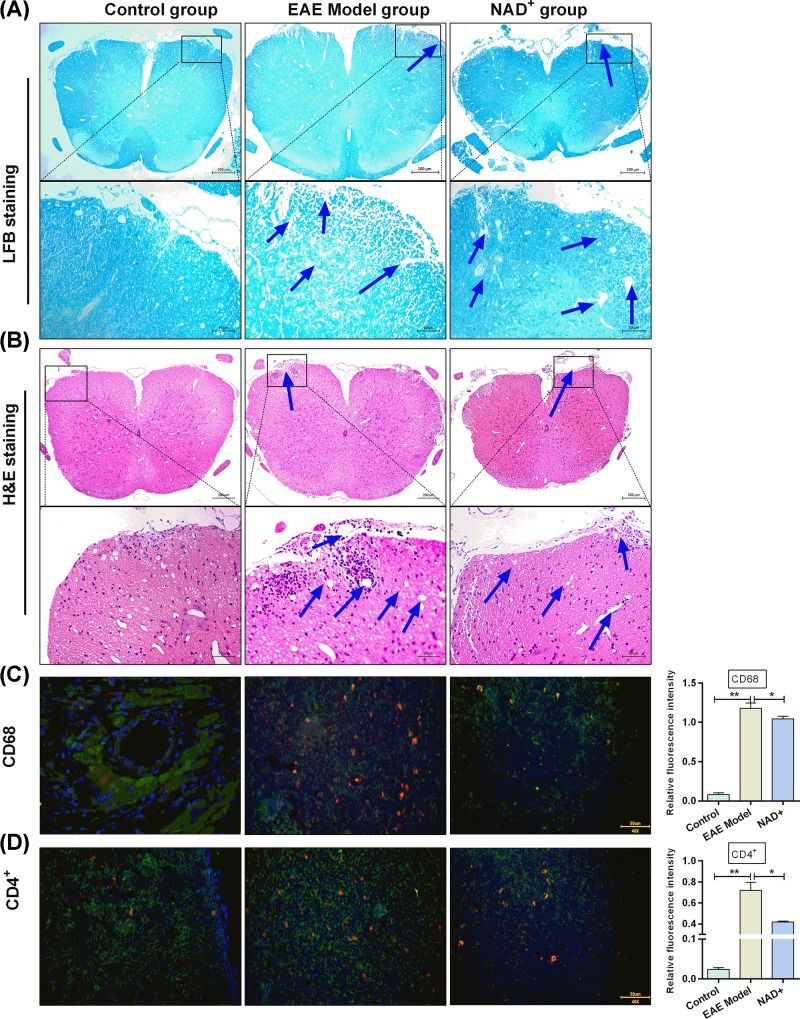
NAD^+^ treatment attenuated EAE (**A**) LFB staining (×200) of the spinal cords in each group. (**B**) HE staining (×200) of mice spinal cords in each group. Blue arrow represented inflammatory cell infiltration. (**C**) Digital images of mice spinal cords’ sections after CD68 immunofluorescence staining and the quantification of the expression of CD68. (**D**) Digital images of mice spinal cords sections after CD4^+^ immunofluorescence staining and the quantification of the expression of CD4^+^. Data presented were the mean ± standard deviation (*n*=10 mice/group). *P<0.05, **P<0.001.

To evaluate the effects of NAD^+^ on EAE, C57BL/6 mice were used to construct EAE models. After construction of EAE models, LFB and Hematoxylin and Eosin (HE) staining were performed to assess inflammation and demyelination, respectively. As shown in Figure 1A,B, a significant increase in demyelination foci and inflammatory cells were observed in the EAE model mice compared with normal mice. In addition, the numbers of macrophage and CD4^+^ T lymphocytes were significantly enhanced in the EAE model mice compared with normal mice (all *P*<0.001) (Figure 1C,D). These results showed the successful construction of EAE models.

**Figure 2:**
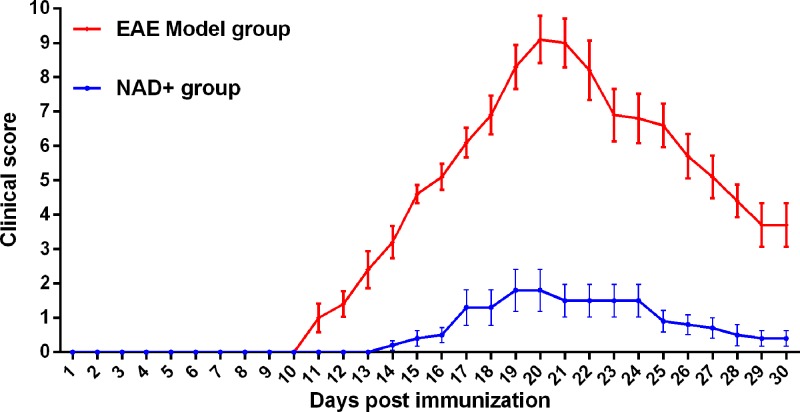
Clinical scores of NAD^+^ and EAE group In each experiment, disease incidence was 100% in EAE group and 60% in NAD^+^ group (*n*=10 mice/group).

After EAE models were treated with NAD^+^, we observed that the significant increase in demyelination foci and inflammatory cells in the spinal cords of EAE model mice were attenuated by NAD^+^ treatment (Figure 1A,B). In addition, NAD^+^ treatment significantly reduced the elevated macrophage and CD4^+^ T lymphocytes numbers in EAE model mice (all *P*<0.05) (Figure 1C,D). We observed that the onset of clinical signs in NAD^+^ group was delayed significantly (Figure 2). The mean day of onset for NAD^+^ group was 16.5 days post-EAE induction, whereas the EAE model group exhibited clinical symptoms as early as day 10, with mean onset at 12.3 day post-immunization. Taken together, the results indicated that NAD^+^ treatment could decrease inflammatory cells and demyelination foci, attenuate the clinical scores of EAE and slightly delaydisease onset.

### NAD^+^ treatment activated the phosphorylation of STAT6 and suppressed NF-κB pathway

**Figure 3:**
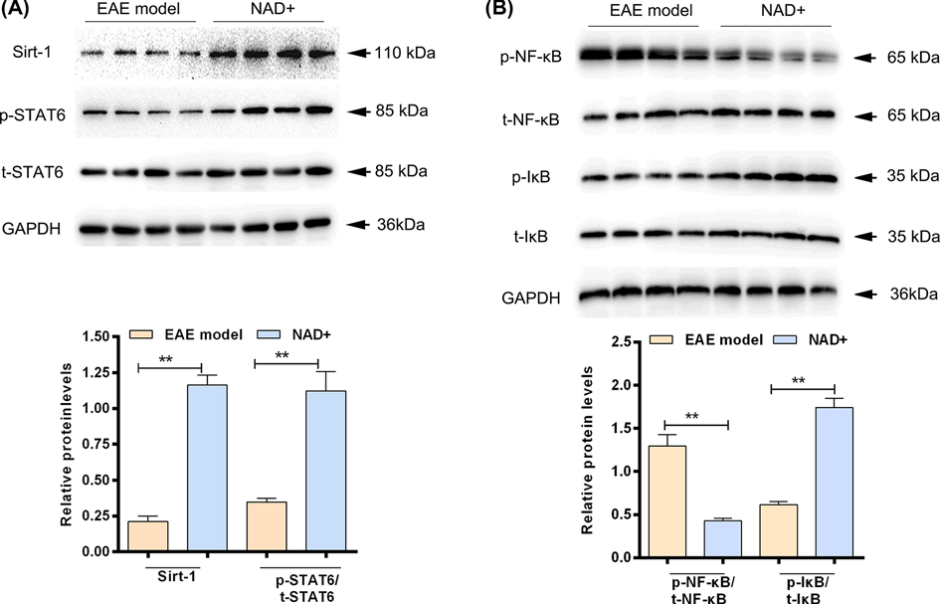
NAD^+^ treatment activated the p-STAT6 expression and suppressed NF-κB pathway in spinal cord (**A**) Western blot analysis of p-STAT6 and SIRT1 expression in each group. (**B**) Western blot analysis of p-NF-κB and p-IκB expression in each group. Data presented were the mean ± standard deviation. **P<0.001.

In order to illuminate the possible mechanisms of NAD^+^ on EAE, we next examined the effects of NAD^+^ on STAT6 and NF-κB in spinal cord. The results showed that the expression level of SIRT-1 and p-STAT6 in NAD^+^ group were significantly higher than that in EAE model group (*P*<0.05) (Figure 3A). In addition, NAD^+^ treatment up-regulated the expression of p-IκB and down-regulated the expression of p-NF-κB (Figure 3B). These results indicated that NAD^+^ treatment could activate the phosphorylation of STAT6 and suppressed NF-κB pathway in EAE.

### NAD^+^ treatment induced CD11b^+^ gr-1^+^ MDSCs

**Figure 4:**
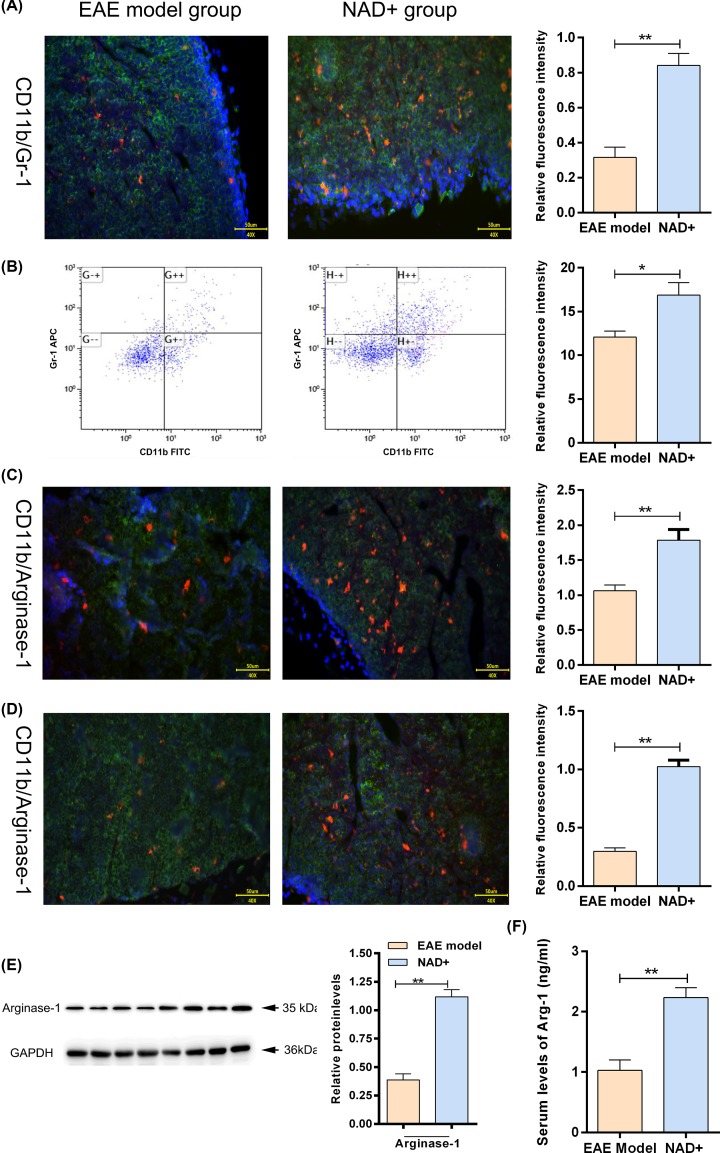
NAD^+^ treatment increased the number of CD11b^+^ gr-1^+^ MDSCs in spleen (**A**) Digital images of mice spinal cords’ sections after CD11b and Gr-1 immunofluorescence staining and the quantification of the expression of CD11b and Gr-1. Green fluorescence represented CD11b and red fluorescence represented Gr-1. (**B**) Splenocytes were stained with CD11b and Gr-1 Ab and then analyzed by flow cytometry. Representative histograms shown. (**C**) Digital images of mice spinal cords’ sections after Arginase-1 immunofluorescence staining and the quantification of the expression of arginase-1. Green fluorescence represented CD11b and red fluorescence represented Arginase-1. (**D**) Digital images of mice spleen sections after arginase-1 immunofluorescence staining and the quantification of the expression of arginase-1. (**E**) Western blot analysis of Arginase-1 expression in spinal cord. (**F**) Serum expression level of Arginase-1 analyzed by ELISA (*n*=10 mice/group). Data presented were the mean ± standard deviation. *P<0.05, **P<0.001.

We examined the numbers of CD11b^+^ gr-1^+^ MDSCs in spleen of each group by immunofluorescent staining and flow cytometry. The results showed that the numbers of CD11b^+^ gr-1^+^ MDSCs in NAD^+^ group were significantly higher than that in EAE model group (*P*<0.05) (Figure 4A,B). The expressions of Arginase-1 were measured using immunofluorescent staining in the spinal cord and spleen of mice. As expected, we found that NAD^+^ treatment significantly enhanced the expressions of Arginase-1 (*P*<0.05) (Figure 4C,D). In addition, Western blot (Figure 4E) and ELISA (Figure 4F) also showed that NAD^+^ treatment significantly enhanced the expressions of Arginase-1 in spinal cord and serum, respectively. These results indicated that NAD^+^ treatment could induce CD11b^+^ gr-1^+^ MDSCs.

### NAD^+^ treatment regulated inflammatory response of Th1, Th2 and Th17 cells

**Figure 5:**
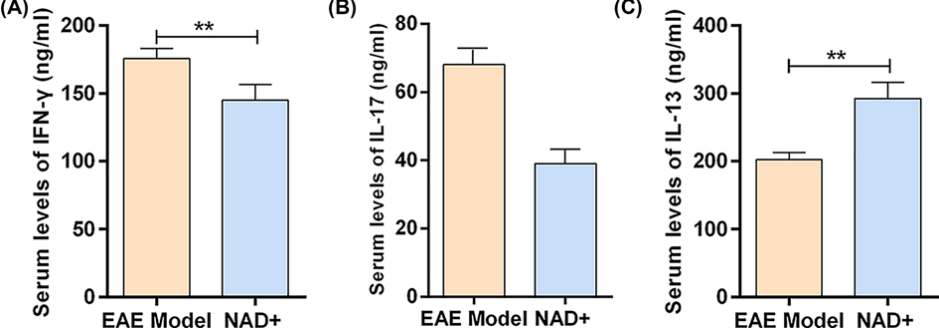
NAD^+^ treatment regulated inflammatory response of Th1, Th2 and Th17 cells (**A**) Serum expression level of IFNγ analyzed by ELISA (*n*=10 mice/group). (**B**) Serum expression level of IL-17 analyzed by ELISA (*n*=10 mice/group). (**C**) Serum expression level of IL-13 analyzed by ELISA (*n*=10 mice/group). Data presented were the mean ± standard deviation. *P<0.05, **P<0.001.

We also characterized inflammatory cytokine expression in each group. Serum collected from EAE model mice displayed elevated levels of IFN-γ and IL-17 while NAD^+^ treatment significantly reduced these inflammatory cytokine levels (Figure 5A,B). Moreover, the expressions of IL-13 in serum of NAD^+^ group were significantly higher than that in EAE model group (Figure 5C). These results indicated that NAD^+^ treatment regulated inflammatory response of Th1 and Th2 cells.

## Discussion

MS is a chronic immune-mediated disease of the CNS [21]. Current treatments for MS are based on anti-inflammation, which unfortunately are only partially effective [4]. Thus, developing novel therapeutic strategies has been an urgent problem for patients with MS. SIRT1 is an NAD^+^-dependent protein deacetylase that catalyzes the removal of acetyl groups from a variety of protein substrates [12]. Previous study showed that SIRT1 induction can attenuate the course of EAE [13]. However, the underlying mechanism has not been well elucidated. In the present study, we established an EAE model and investigate the possible mechanisms of NAD^+^ in attenuating the course of EAE. The highlights of present study are the following: first, we demonstrated that NAD^+^ treatment could activate the phosphorylation of STAT6. Second, we demonstrated that NAD^+^ treatment induced CD11b^+^ gr-1^+^ MDSCs. Third, we found that NAD^+^ treatment regulated inflammatory response of Th1 and Th2 cells. Finally, we proposed a novel mechanism for NAD^+^ attenuate EAE, in which NAD^+^ treatment may induce the CD11b^+^ gr-1^+^ MDSCs to attenuate EAE via activating the phosphorylation of STAT6 expression.

In the present study, we first verified the effect of NAD^+^ on the course of EAE. The results showed that NAD^+^ treatment could attenuate the clinical scores of EAE, slightly delay disease onset and decrease inflammatory cells and demyelination foci. Similarly, Tullius et al. [19] demonstrated that NAD^+^ could block EAE by inducing immune homeostasis via CD4^+^IFNγ^+^IL-10^+^ T cells and reverse disease progression by restoring tissue integrity through neuroregeneration and remyelination. Besides, the study performed by Wang et al. [13] had indicated that NAD^+^ treatment attenuated pathological injuries of EAE mice. Although studies reported the effects of NAD^+^ in EAE, underlying mechanism remained controversial. Thus, we further investigated possible mechanism of NAD^+^ treatment attenuated EAE.

STAT6 is a member of the STAT family and plays important roles in cell differentiation and immune responses induced by growth factors, cytokines and other cell activators [22]. Previous study showed that SIRT1 plays an anti-inflammatory role through the PI3K/Akt/STAT6 pathway in acute gout [14]. However, the regulatory effect of SIRT1 on STAT6 in EAE remains unclear. Therefore, the present study investigated the effects of NAD^+^ on STAT6 in EAE model. Interestingly, the results illuminated that NAD^+^ treatment could activate the phosphorylation of STAT6. p-STAT6 was observed in intestinal epithelial cells of patients with inflammatory bowel disease [23]. Thus, the above findings indicated that NAD^+^ treatment may attenuate EAE via activating the phosphorylation of STAT6 expression.

MDSCs are immunosuppressive cells which could inhibit acute inflammatory reactions, trigger the resolution of inflammation and initiate the repair processes [24]. Recent studies have shown that MDSCs play important role in shaping T-cell responses [25,26]. MDSCs suppressed T-cell responses by several mechanisms including the production of IL4-dependent arginase [27,28]. Our studies demonstrate that NAD^+^ treatment increased the numbers of CD11b^+^ gr-1^+^ MDSCs and the expression of Arginase-1. Although, several studies demonstrated that MDSCs could attenuate the clinical course of EAE [9,10]. STAT6 was reported to promote the expansion of MDSCs in the lamina propria and spleen of ApcMin/^+^ mice [11]. However, the regulatory effect of STAT6 on MDSCs in EAE remains unclear.

MDSCs used inducible nitric oxide synthase (NOS2) and Arginase-1 to control T-cell responses [29]. Th1 cytokines (such as IFN-γ and TNF-α) induce NOS2, whereas Th2 cytokines (such as IL-4 and IL-13) up-regulate Arginase-1 [30]. In order to verify our hypothesis, we further characterized inflammatory cytokine expression in NAD^+^ and EAE model groups. The results showed that NAD^+^ treatment regulated inflammatory response of Th1 and Th2 cells and promote IL-13 and inhibit IFN-γ secretion in EAE mice. These findings indicated that NAD^+^ treatment may attenuate EAE by induction of CD11b^+^ gr-1^+^ MDSCs via regulating inflammatory response of Th1 and Th2 cells. More importantly, studies reported that STAT6 was reported to be involved in the regulation of the Th1/Th2 immune response [31]. This indicated that NAD^+^ treatment may attenuate EAE by regulating inflammatory response of Th1 and Th2 cells via activating the phosphorylation of STAT6. Taken together, our studies demonstrate that NAD^+^ treatment may attenuate EAE by inducing of CD11b^+^ gr-1^+^ MDSCs via activated the phosphorylation of STAT6.

## Conclusions

The present study further confirmed the effects of NAD^+^ on the pathogenesis of EAE. Our studies demonstrate that NAD^+^ treatment attenuated the course of EAE and activated the phosphorylation of STAT6 expression. Additionally, NAD^+^ treatment induced CD11b^+^ gr-1^+^ MDSCs and inhibited inflammatory responses by regulating Th1, Th2 and Th17 cells. Taken together, our studies demonstrated that NAD^+^ treatment may attenuate EAE by inducing of CD11b^+^ gr-1^+^ MDSCs via activated the phosphorylation of STAT6. Therefore, NAD^+^ may be considered as a potential novel therapeutic strategy for MS.

## Abbreviations

CNS: central nervous system
EAE: experimental autoimmune encephalomyelitis
ELISA: enzyme-linked immunosorbent assay
IFN-γ: interferon γ
IκB: inhibitor of kappa B
IL: interleukin
LFB: Luxol Fast Blue
MDSC: myeloid-derived suppressor cell
MS: multiple sclerosis
NAD^+^: nicotinamide adenine dinucleotide
NOS2: nitric oxide synthase
PBS: phosphate buffer saline
PVDF: polyvinylidene fluoride
p-STAT6: phosphorylated-STAT6
SIRT1: sirtuin 1
